# Interpretation of the concept ‘nursing’: Utilisation in nursing education and practice

**DOI:** 10.4102/curationis.v45i1.2351

**Published:** 2022-11-29

**Authors:** Sundira D. Mottian, Lizeth Roets, Kefiloe A. Maboe

**Affiliations:** 1 Department of Health Studies, College of Human Sciences, University of South Africa, Pretoria, South Africa

**Keywords:** Concept analysis, faculty nursing, nurses, nursing, nurse practitioner, nursing service, student nurses

## Abstract

**Background:**

Nursing developed over centuries. Changing practice and education influenced its interpretation and understanding. Its meaning and interpretation may differ amongst education institutions, nurse educators and nurses, particularly student nurses.

**Objectives:**

The objective was to develop a visual concept map of the concept of ‘nursing’, allowing nursing education institutions to have a similar approach and understanding in teaching the concept to student nurses.

**Method:**

The research design was qualitative, explorative, descriptive and contextual. A self-designed, pretested online questionnaire collected data from various categories of nurse participants. An integrative review viewed literature sources published between 2006 and 2016 accessing definitions of ‘nursing’. Data analysis involved thematic analysis of narrative data, data coding processes, interpretation and synthesis of data and further analysis using a systematic concept analysis process. The combined analysed data merged, developing a visual concept map of ‘nursing’. Expert nurse educators validated the visual concept map of ‘nursing’ by e-Delphi technique, using an assessment rubric.

**Results:**

Various definitions of ‘nursing’ revealed identified themes and categories underpinning the concept. After formulation of connotative, denotative definitions and empirical referents, a visual concept map of ‘nursing’ was developed and validated to be an educational tool to facilitate the teaching of the concept of ‘nursing’, enhancing a similar understanding and interpretation thereof.

**Conclusion:**

A visual concept map of ‘nursing’, a tool facilitating teaching the concept and promoting similar understanding of its meaning is valuable in an evolving digital era, where visual stimulation enhances teaching and learning.

**Contribution:**

The primary contribution of the manuscript provided a developed visual concept map of ‘nursing’, to use as a tool to stimulate critical thinking and integrate the various aspects outlined in the map. The visual concept map of ‘nursing’ assists in the education and training of all categories of nurses in the profession, especially student nurses, aiming to support better patient outcomes when the concept of nursing is understood and interpreted in the same way.

## Introduction

Literature sources express various definitions of the concept of ‘nursing’. Florence Nightingale in the 19th century defined nursing as: ‘putting the patient in the best condition for nature to act upon him’ (Nightingale 1860:133). In the 20th century, other definitions were: ‘nursing is a process that was serial and goal-directed demanding certain steps, actions, operations or performances that occurred between the nurse and the person who was nursed’ (Peplau 1952). Nursing was also defined as ‘to assist the individual, sick or well, in the performance of those activities contributing to health or its recovery (or a peaceful death)’ (Henderson 1964).

More recent definitions of ‘nursing’ are:

The use of clinical judgment in the provision of care to enable people to improve, maintain, or recover health, to cope with health problems, and to achieve the best possible quality of life, in the case of disease or disability, until death. (Royal College of Nursing 2003:3)

The South African nursing regulatory body, namely the South African Nursing Council (SANC), define nursing as:

[*A*] caring profession practiced by a person registered with the SANC, who supports, cares for and treats a healthcare user to achieve or maintain health. Where this is not possible, a nursing professional cares for a healthcare user so that they live in comfort and with dignity until death. (SANC Nursing Act 2005:6)

The International Council of Nurses (2002) expresses nursing as:

[*N*]ursing includes the promotion of health, prevention of illness, and care of the sick, disabled and dying people, advocacy, promotion of a safe environment, research, participation in shaping health policy and in patient and health systems’ management. Nursing is about the privilege of being able to share in the joys and sorrows of people’s lives and making a difference. It is empowering people to make decisions about their health, birth and death and supporting them in their choices. Nursing is about talking, teaching, touching, smiling and crying. Nursing is caring. (Howie & Robertson 2017:i)

Over the years, with the evolution of nursing, the definitions of nursing have transformed. The definition expanded with advancement in technology and science. In view of this, the SANC, a statutory body governing the practice of nursing in South Africa, provides a definition of nursing encompassed in *Act No. 33 of 2005*: *Nursing Act, 2005*. It can be suggested that since nurses and nurse educators are being guided by legislative entities, revisiting the definition should be considered, taking into account the radical evolution of nursing practice. However, on the contrary, arguably the definition of nursing provided by the SANC may be considered a standardising definition, as nursing will always require basic care rendered to the healthcare user despite all the advancement in technology and science. Therapeutic care is required despite working with complex technology. Much of the nurses’ work still involves everyday domestic tasks, emotional support and environmental control. Nurses have to feed patients, bathe them, ensure beds are made with fresh linen, open windows for fresh air and comfort those who need reassurance (Keeling, Hehman & Kirchgessner 2018:160).

The various definitions of ‘nursing’ create an opening for individual nurses and nurse educators to have a different understanding and interpretation of the concept. The different interpretations of the concept of ‘nursing’ influence the way in which nurses think about what nursing actually entails and seemingly affects the outcome of nursing care provided to the healthcare consumers. The foundation of how nursing is practised stems from the interpretation and understanding of what nursing means. A similar understanding and interpretation of the concept ultimately reduces the healthcare consumer’s exposure to risk of harm when under the care of nurses. The concept map of ‘nursing’ will be a guideline, utilised by nurse educators when teaching the concept of ‘nursing’. This tool will facilitate a common interpretation and understanding of the concept, thus promoting the practice of nursing in the same way to benefit all healthcare consumers when the key attributes of the concept are implemented clinically.

The study aimed to develop a visual concept map of ‘nursing’, a tool to facilitate nurse educators during the teaching of the concept of ‘nursing’. The concept map is a benchmark to promote the similar understanding, meaning and interpretation of ‘nursing’, thereby ensuring the practice of nursing in the same way when the key attributes of the concept are expressed when in contact with the healthcare consumers.

## Theoretical framework

The theoretical framework adopted was Wilson’s method of concept analysis (1963), which was revised to an eight-step concept analysis process by Walker and Avant (2011). Concept analysis is a strategy that identifies a set of characteristics essential to defining the connotative meaning of a concept (Gray & Grove 2020:173). The eight steps of the concept analysis process were: (1) select a concept, (2) determine the aims or purposes of analysis, (3) identify all uses of the concept, (4) determine the defining attributes, (5) identify a model case, (6) identify a contrary case, (7) identify antecedents and consequence and (8) define empirical referents. These systematic steps facilitated the critical analysis of the meaning and interpretation of the concept of ‘nursing’ and promoted clarification of its meaning.

## Research methods and design

### Objectives

The objectives of the study were to: (1) explain the meaning and interpretation of the definition of ‘nursing’ from different categories of nurses registered with SANC and as members of Democratic Nursing Organisation of South Africa (DENOSA); (2) provide evidence for the development of a visual concept map that can be utilised by nursing education institutions in teaching the concept.

### Research design

**FIGURE 1:**
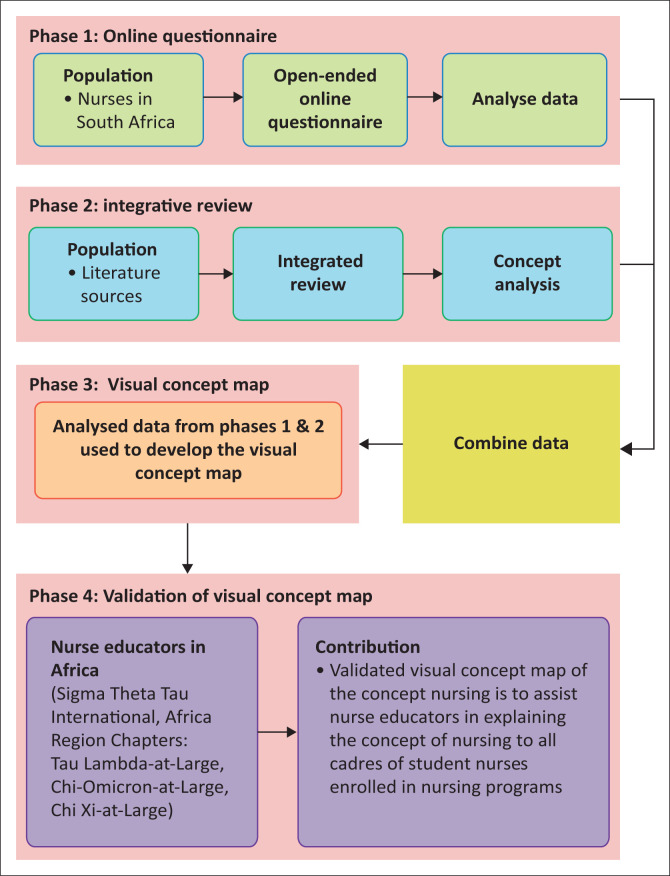
Research phases.

A qualitative, explorative, descriptive and contextual approach facilitated detailed analysis of the concept of ‘nursing’ and the development of the visual concept map of ‘nursing’. Figure 1 illustrates the research phases.

### Setting and population

The study was conducted in four phases. The setting and population for each phase were as follows: Phase 1 included nurse participants from various categories who were registered with SANC and were members of DENOSA, a total of 84 000 (DENOSA 2022:1). In Phase 2, the integrative review, the population comprised any online or hard copies of English books, encyclopaedias, journals, newspaper articles, published and unpublished theses and articles, from any discipline containing the definition of ‘nursing’, published between 2006 and 2016. Phase 3 included gathered data from Phases 1 and 2, and finally in Phase 4, the nurse educators inaugurated in Sigma Theta Tau International (STTI), the Africa Region Chapters (Tau Lambda at-Large, Chi Omicron at-Large and Chi Xi at-Large Chapters), formed the setting and the population.

### Sampling

The study followed a nonrandom sampling approach. A selected heterogeneous sample (nurses), *n* = 415, formed the sample for Phase 1, in which participants took an online survey. Various literature sources specifically chosen with publication from 2006 to 2016 were the sample in Phase 2. An all-inclusive sampling during Phase 3 developed the concept map of ‘nursing’ using all data collected from Phases 1 and 2. Phase 4 sampling included specifically chosen nurse educators, directly involved in updating and improving nursing and nursing education within Africa and teaching the concept of ‘nursing’ to student nurses.

### Data collection

**FIGURE 2:**
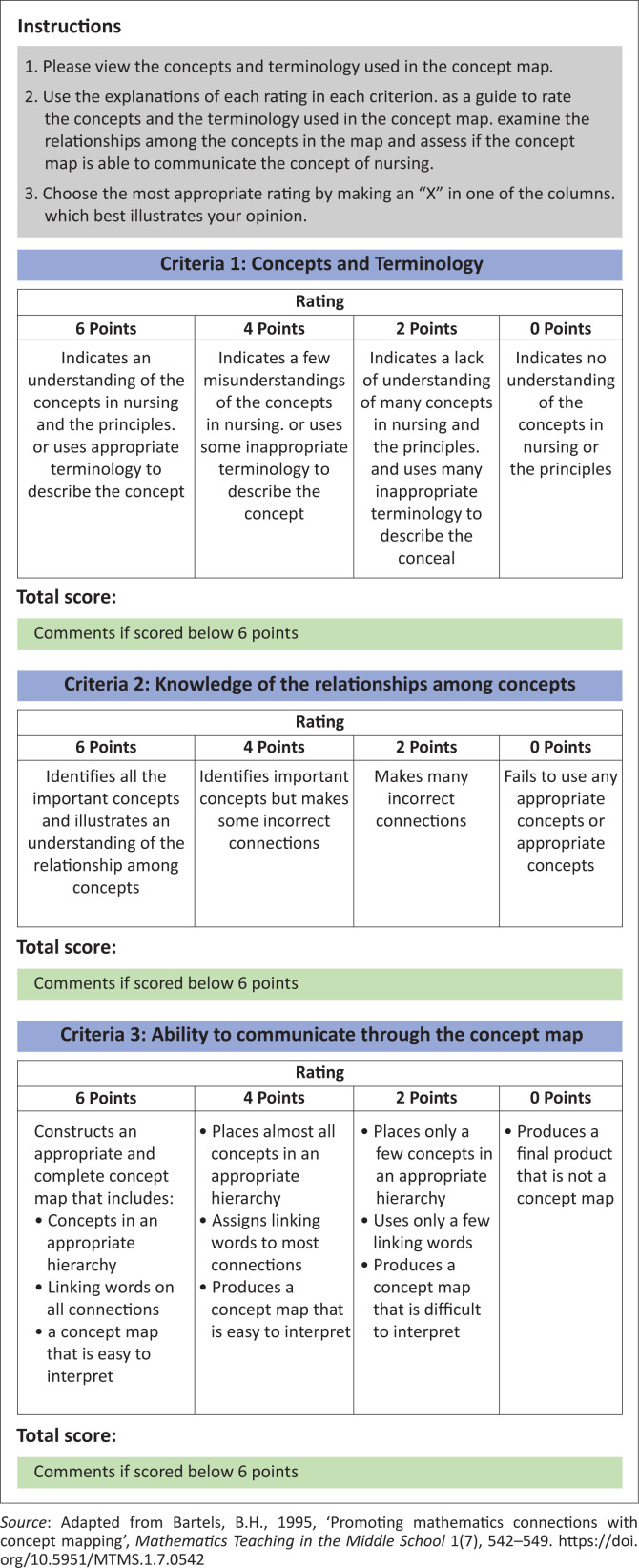
Validation assessment rubric (Bartels 1995:545).

Data were collected from 19 March 2017 to 19 June 2017. An online pretested questionnaire via SurveyMonkey™ (Momentive, Inc., San Mateo, California, United States) facilitated data collection in Phase 1. An invitation recruitment letter, including a web link address (https://forms.gle/oovijxebU2ueAp7X6) to access the online questionnaire was sent to all nurses registered on the DENOSA database. Participants voluntarily clicked on the web link address accessing the online questionnaire, and on clicking the ‘NEXT’ button, they voluntarily agreed to participation to the study. Questionnaire completion was over a period of three months, with reminder e-mails sent fortnightly. All ethical considerations related to informed consent, participation, confidentiality and anonymity were applied. During Phase 2, an integrative review pursued consulting related literature sources from 2006 to 2016, with definitions of ‘nursing’. The merged data from Phases 1 and 2 resulted in the development of a visual concept map of nursing in Phase 3. Finally, in Phase 4, the heads of department of schools of nursing of universities belonging to Africa Region Chapters of STTI were e-mailed, requesting three randomly selected nurse educators inaugurated in STTI to participate in the validation process of the visual concept map of ‘nursing’. Participants responded to the e-mail request and invitation, implying acceptance to participation. The visual concept map of nursing, the assessment rubric (see Figure 2) and instructions for its use were accessed by participants via a web link address.

### Data analysis

Data from 415 questionnaires were analysed using the SurveyMonkey™ data analysis software. Participants’ demographic data were not fundamental to the study; however, their context could enhance transferability of data to similar context. Data from the open-ended questions related to participants’ input on the concept of ‘nursing’ followed thematic analysis of the narrative data, which was coded according to Tesch’s (1992) coding guidelines.

The integrative review and data analysis used step 4 of integrative reviews, interpretation and synthesis of the data, outlined by Whittemore and Knafl (2005:550–551). Further analysis of the data included the adapted eight-step concept analysis process by Walker and Avant (2011).

The combined analysed data from Phases 1 and 2 formulated a visual concept map of nursing arranged in a spider map style, following Novak and Canas’s (2006:1–2) steps to develop a concept map.

Finally, the developed visual concept map of nursing was validated by seven experts in nursing education, using an e-Delphi technique during two rounds of validation when over 75% consensus was reached between participants. An assessment rubric by Bartels (1995:545), outlined in Figure 2, facilitated the validation process.

### Ethical considerations

Ethical approval to conduct this study was obtained from the University of South Africa Health Studies Higher Degrees Committee College of Human Sciences (ref. no. HSHDC/486/2015, REC-012714-039).

## Results

After data coding and analysis, emerged categories were (1) ‘altruism’, (2) ‘caring holistically’, (3) ‘cognitive abilities’, (4) ‘collaboration’, (5) ‘compassionate’, (6) ‘dedication’, (7) ‘dignity’, (8) ‘dynamism’, (9) ‘health promotion’, (10) ‘legislature’, (11) ‘meeting needs’, (12) ‘noble’, (13) ‘profession’, (14) ‘research’, (15) ‘support’ and (16) ‘unbiased’. The exposed themes were (1) affective, (2) cognitive and (3) social and behaviour.

**FIGURE 3:**
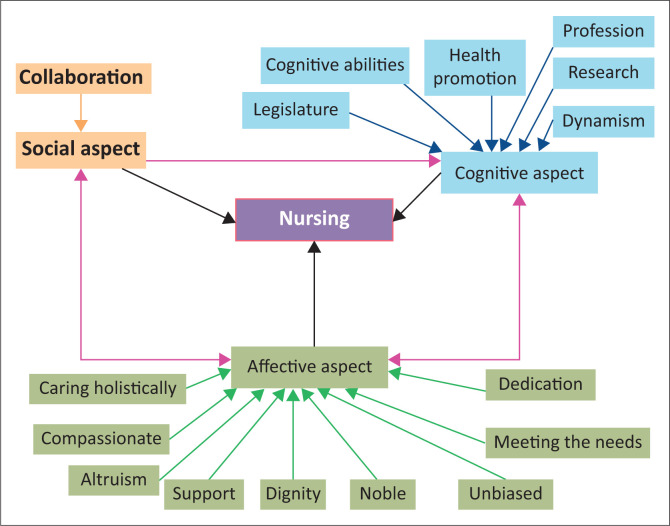
Visual concept map of nursing.

In the developed concept map, the concept of ‘nursing’ was the main point of focus. The categories identified were the key attributes of the concept of ‘nursing’, and hierarchical relations between concepts were illustrated. The spider map style reveals the concept of ‘nursing’ at the centre of the spider map, with the themes and categories and subcategories branching towards the centre expressing the importance of the main concept. The linking arrows connect key attributes (categories) of nursing, depicting the relationships between the themes: affective, cognitive and social aspects (see Figure 3).

Participants expressed suggestions to enhance the concept map during rounds of validation. There was uncertainty about the term ‘dynamism’ and deficiency in terms of the behaviour aspect; the psychomotor skills aspect had shortfalls, and ‘meeting needs’ should have been included in the social and cognitive parts of the concept map; finally, the ethical code of conduct needed to be addressed.

**FIGURE 4:**
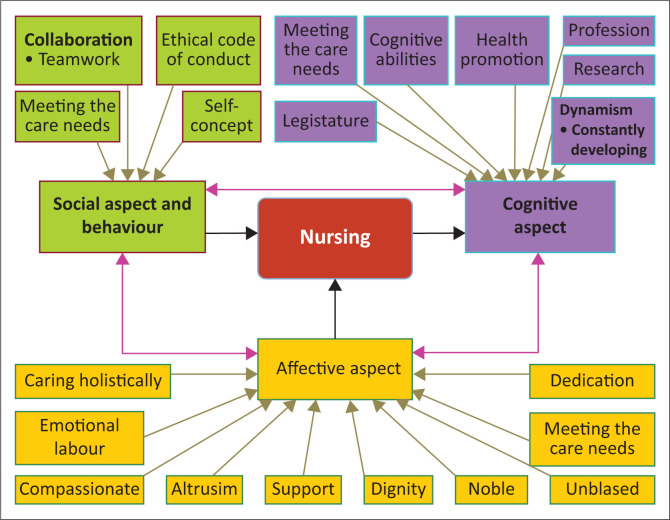
Final concept map of nursing.

All participants’ comments were viewed and qualitatively analysed according to the guidelines by Tesch (1992), with necessary amendments to the visual concept map. The raw data were reviewed to facilitate amendments, and the final concept map of nursing (see Figure 4) was accepted as validation was completed, after a consensus of 86% was reached between the panellists.

## Discussion

The concept map of nursing contributes a visual presentation to facilitate the teaching of the concept of ‘nursing’. The visual map was designed to assist student nurses to learn in a more meaningful way with the aim of having a similar interpretation and understanding of the concept of ‘nursing’. The layout of the concept map is visual and clear to facilitate understanding of the structure of the concept of ‘nursing’. Teaching becomes highly interactive when student nurses have the opportunity to engage with key attributes in the visual concept map to stimulate critical thinking that can progress into a discussion of the meaning of ‘nursing’. Nurse educators can direct the student nurse’s attention to the inter-relationships of the different aspects of the concept as depicted by the linking arrows. Concerns and expressions about the definition are encouraged to assist with clarity, ensuring a similar interpretation and understanding of the concept of ‘nursing’.

Possibly, pre-exposure to the definition of nursing as outlined by the SANC could have influenced nurses to tailor their definition of the concept of ‘nursing’. Many times, more effort is required for new thoughts and ideas about a topic, a new definition or understanding of a concept as opposed to relying on the familiar. A visual concept map to facilitate the understanding of the concept of nursing can therefore contribute to similar understanding and new insights to the concept.

## Conclusion

A tool was developed to teach the concept of nursing to students so that it can be interpreted and understood in the same way. The visual concept map of nursing was developed after analysing various definitions of the concept provided by nurses as well as various literature sources. The concept map is an asset to the education process as visual concept maps stimulate critical thinking and participation of students in their learning.
